# Community seroprevalence of SARS-CoV-2 in children and adolescents in England, 2019–2021

**DOI:** 10.1136/archdischild-2022-324375

**Published:** 2022-07-20

**Authors:** Helen Ratcliffe, K S Tiley, Nick Andrews, Gayatri Amirthalingam, I Vichos, E Morey, N L Douglas, S Marinou, Emma Plested, Parvinder Aley, Eva P Galiza, Saul N Faust, S Hughes, Clare S Murray, Marion Roderick, Fiona Shackley, Sam J Oddie, Tim Lees, D P J Turner, M Raman, Stephen Owens, Paul Turner, H Cockerill, J Lopez Bernal, E Linley, Ray Borrow, Kevin Brown, Mary Elizabeth Ramsay, M Voysey, Matthew D Snape

**Affiliations:** 1 Department of Paediatrics, University of Oxford, Oxford, UK; 2 Statistics, Modelling and Economics Department, Health Protection Agency, London, UK; 3 Immunisation, Hepatitis and Blood Safety Department, Public Health England, London, UK; 4 St George's Vaccine Institute, St. George’s University Hospitals NHS Foundation Trust, London, UK; 5 Academic Unit of Clinical & Experimental Sciences, Faculty of Medicine, University of Southampton, Southampton, UK; 6 NIHR Wellcome Trust Clinical Research Facility, University Hospital Southampton NHS Foundation Trust, Southampton, UK; 7 Department of Paediatrics, Royal Manchester Children’s Hospital, Manchester, UK; 8 Respiratory Group, University of Manchester, Manchester, UK; 9 Paediatric Infectious Diseases and Immunology, University Hospitals Bristol NHS Foundation Trust, Bristol, UK; 10 Immunology, Allergy and Infectious Diseases, Sheffield Children's Hospital NHS Foundation Trust, Sheffield, UK; 11 Bradford Neonatology, Bradford Teaching Hospitals NHS Foundation Trust, West Yorkshire, UK; 12 Paediatric Respiratory Medicine, Leeds Teaching Hospitals NHS Trust, Leeds, UK; 13 School of Life Sciences, University of Nottingham and Nottingham University Hospitals NHS Trust, Nottingham, UK; 14 Department of Paediatrics, University Hospitals Plymouth NHS Trust, Plymouth, UK; 15 Paediatric Immunology and Infectious Diseases, Newcastle upon Tyne Hospitals NHS Foundation Trust, Newcastle upon Tyne, UK; 16 Population Health Sciences Institute, Newcastle University, Newcastle upon Tyne, UK; 17 Section of Paediatrics, Imperial College London, London, UK; 18 Department of Paediatrics, West Suffolk NHS Foundation Trust, Bury Saint Edmunds, UK; 19 Vaccine Evaluation Unit, UK Health Security Agency, London, UK; 20 Virus Reference Department, Public Health England, Colindale, UK

**Keywords:** COVID-19, epidemiology, healthcare disparities, paediatrics

## Abstract

**Objective:**

To understand community seroprevalence of SARS-CoV-2 in children and adolescents. This is vital to understanding the susceptibility of this cohort to COVID-19 and to inform public health policy for disease control such as immunisation.

**Design:**

We conducted a community-based cross-sectional seroprevalence study in participants aged 0–18 years old recruiting from seven regions in England between October 2019 and June 2021 and collecting extensive demographic and symptom data. Serum samples were tested for antibodies against SARS-CoV-2 spike and nucleocapsid proteins using Roche assays processed at UK Health Security Agency laboratories. Prevalence estimates were calculated for six time periods and were standardised by age group, ethnicity and National Health Service region.

**Results:**

Post-first wave (June–August 2020), the (anti-spike IgG) adjusted seroprevalence was 5.2%, varying from 0.9% (participants 10–14 years old) to 9.5% (participants 5–9 years old). By April–June 2021, this had increased to 19.9%, varying from 13.9% (participants 0–4 years old) to 32.7% (participants 15–18 years old). Minority ethnic groups had higher risk of SARS-CoV-2 seropositivity than white participants (OR 1.4, 95% CI 1.0 to 2.0), after adjusting for sex, age, region, time period, deprivation and urban/rural geography. In children <10 years, there were no symptoms or symptom clusters that reliably predicted seropositivity. Overall, 48% of seropositive participants with complete questionnaire data recalled no symptoms between February 2020 and their study visit.

**Conclusions:**

Approximately one-third of participants aged 15–18 years old had evidence of antibodies against SARS-CoV-2 prior to the introduction of widespread vaccination. These data demonstrate that ethnic background is independently associated with risk of SARS-CoV-2 infection in children.

**Trial registration number:**

NCT04061382.

## Abstract (precis)

How many children have antibody to COVID-19 before they are vaccinated? This community seroprevalance study finds about a third of children with antibody to COVID (although this varies by age and ethnicity). In children <10-years there were no symptoms or symptom clusters that reliably predicted seropositivity.

**What is already known on this topic**

- Previous serostudies show children are frequently asymptomatic or have mild symptoms of COVID-19 infection.
- Ancestral lineages A and B presented predominantly with gastrointestinal symptoms in the paediatric population.
- Minority ethnic groups are at increased risk of seropositivity for SARS-CoV-2.

**What this study adds**

- Community-based recruitment of participants aged 0–18 years old representative of seven National Health Service regions allowing generalisations to be made across England as a whole.
- Approximately one-third of participants 15–18 years old had evidence of antibodies against SARS-CoV-2 prior to the introduction of widespread immunisation in June 2021.
- In children <10 years, there were no symptoms or symptom clusters that reliably predicted seropositivity.

**How this study might affect research, practice or policy**

- Seroprevalence studies provide estimates of the levels of immunity within the paediatric population, vital for modelling of disease susceptibility and immunisation planning in England.
- This study creates a unique biobank from children and young adults aged 0–24 years with a comprehensive history of immunisation and demography for future research.

## Introduction

Seroprevalence studies evaluating population prevalence of SARS-CoV-2 antibodies have an important role in understanding the spread of and population vulnerability to SARS-CoV-2 infection. The majority have been performed in adults,^1 2^ and those in children have predominantly tested samples obtained opportunistically in a clinical context or in school-based populations,^3–5^ both of which bring potential biases.

In response to the COVID-19 pandemic, the Oxford Vaccine Group, in collaboration with UK Health Security Agency, modified an existing seroprevalence pilot study (‘What’s the STORY?’) evaluating serum antibody concentrations against vaccine preventable diseases to determine anti-SARS-CoV-2 serum antibodies across England. This study was funded by the National Institute for Health Research.

Here we report SARS-CoV-2 sero-epidemiology in participants aged 0–18 years old in England prior to widespread immunisation in this population from samples collected from the end of 2019 through to mid-2021. In addition, we explore potential risk factors for COVID-19 seropositivity and the utility of symptom-based indicators of infection.

## Methods

### Study design

This was a cross-sectional seroprevalence study recruiting participants from 13 sites distributed across all seven National Health Service (NHS) regions in England, conducted between October 2019 and June 2021. Eleven sites recruited participants aged 0–24 years (data for those aged 0–18 years old presented here) from postcode districts representative of their NHS region in terms of deprivation (defined by 2019 Index of Multiple Deprivation (IMD)^6^) and urban/rural ratio as identified by local knowledge (online supplemental table 1). IMD measures deprivation available at a Lower Super Output Area (LSOA, an area with an average population of 1500) level and based on seven domains of deprivation (income, employment, education, health, crime, barriers to housing and services and living environment).^6^ Invitation letters were sent through Docmail (a UK General Data Protection Regulation compliant bulk mailing system) from extracts provided by either NHS Digital^7^ or Child Health Information Systems (CHIS)^8^ databases, in addition to social media campaigns. Two sites recruited participants aged 0–19 years old via social media campaigns and were not postcode restricted. Potential participants and their families were invited to visit the study website (https://whatsthestory.web.ox.ac.uk) for additional information and local teams’ contact details

10.1136/archdischild-2022-324375.supp1 Supplementary data

Due to emerging differences in COVID-19 infection in minority ethnic groups in adult studies,^1 9^ an enhanced recruitment strategy for ethnic minority groups started in January 2021. Multiple strategies (targeted mail outs, social media, text messages from general practitioners (GPs), pharmacy advertising) were used.

### Data collection

Participants or their parent/guardian recorded (electronically or on a paper form) responses to questions regarding the participant’s demographics, as well as selected questions from the UK Census 2011 relating to accommodation and employment, and from the Family Affluence Scale iii (FASiii)^10^ relating to socioeconomic status. These data were collected using the Research Electronic Data capture system, V.10.6.13. Responses to the individual-level UK Census^11^ and FASiii questions^10^ for those aged 0–15 years old (scored out of a total of 13^12^) were used to assess the appropriateness of the LSOA-level IMD and the Income Deprivation Affecting Children Index (IDACI, the proportion of children aged 0–15 years living in income-deprived families) scores for our study.

In response to the pandemic, from February 2020, questionnaires were adapted to ask if participants and/or their household had experienced any potential COVID-19 symptoms (fever, dry cough, shortness of breath, muscle aches, feeling tired, loss of appetite). This was further adapted in July 2020 to enable description of which symptoms participants and household contacts had experienced, self-reported results of relevant PCR or antibody testing. Those participants who were already enrolled were approached retrospectively to collect missing data from the updated questionnaire (online supplemental appendix 1). Receipt of a COVID-19 vaccine and vaccination date were recorded either at the visit from personal written documentation or afterwards from GP or CHIS records.

### Measurement of serum antibodies

Blood samples were analysed for SARS-CoV-2-specific antibody responses using the Roche Elecsys Anti-SARS-CoV-2 serological assays for the detection of anti-SARS-CoV-2 IgG spike protein (RocheS) and nucleocapsid (RocheN) antibodies in serum/plasma samples using electrochemiluminescent immunoassays.^13 14^ Both assays report high sensitivity and specificity (online supplemental table 2).^13 14^

### Statistical analysis

#### Seroprevalence

**Figure 1:**
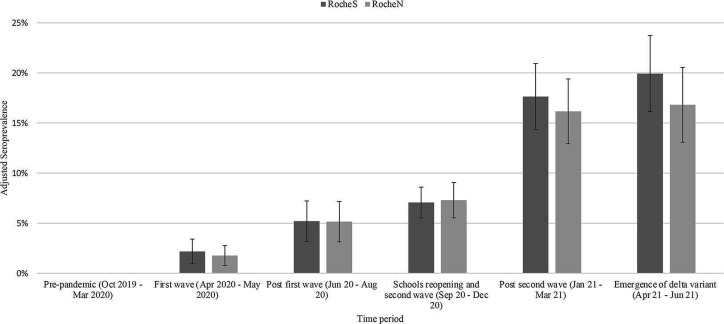
Overall SARS-CoV-2 seroprevalence (RocheS (anti-SARS-CoV-2 IgG spike protein antibodies) and RocheN (anti-SARS-CoV-2 IgG nucleocapsid antibodies)) by time period October 2019–June 2021 in England, adjusted for age, National Health Service region and ethnicity. Error bars indicate 95% CI. (1) First national lockdown came into force (26 March 2020). Schools closed with only children of key workers attending school.^15^ (2) Phased reopening of schools (1 June 2020). Pupils aged 5, 6 and 11 years returned to school. 16- and 18-year-olds were allowed to attend in limited times.^15^ (3) Variant of concern – B.1.1.7 (Alpha) first detected in the UK and sequenced in September 2020.^16^ (4) Second national lockdown came into force (5th November 2020). Schools closed with only children of key workers attending school^16^ (5) Second national lockdown came to an end (2 December 2020)^15^ (6) Variant of concern B.1.351 (Beta) variant first detected in South Africa and was first sequenced in December 2020.^16^ (7) Third national lockdown came into force (6 January 2021). Schools closed with only children of key workers attending school.^15^ (8) Variant of concern – P.1 (Gamma) first detected in Japan in travellers from Brazil in January 2021 and was first detected in the UK in February 2021.^16^ (9) Primary and secondary schools reopen in England (8 March 2021).^15^ (10) Variant of concern B.1.617.2 (Delta) variant first detected in India were first detected in the United Kingdom in mid-April 2021.^16^ All legal limits on social contact removed (21 June 2021).^15^

The unweighted observed prevalence for RocheS and RocheN separately was calculated as n+/N for children aged 0–18 years, where n+ was the number of individuals who tested positive, and N was the total number of individuals tested with an available result. Unweighted prevalence was calculated for each of six time periods (figure 1),^15 16^ overall, by age group (0–4 years, 5–9 years, 10–14 years, 15–18 years) and by region. Prevalence estimates for each time period were standardised by age group, ethnicity and NHS region using the STATA stdize command. Population estimates of demographic variables were determined from NewETHPOP-Evaluation, Revision and Extension of Ethnic Population Projections.^17^

#### Risk factors

Age group, sex, NHS region, time period, ethnicity (grouped as white and minority ethnic groups), IMD or Income Deprivation Affecting Children Index (IDACI) deprivation quintile (for comparison) and urban/rural classifications were analysed in univariate and multivariable logistic regression models. A separate model tested the presence of a healthcare worker in the family as a risk factor.

#### Symptoms

Symptoms associated with seropositivity were explored for participants aged 0–9 years old and 10–18 years old separately to optimise statistical power while allowing discrimination of differences between participants attending early-years and primary school educational settings compared with secondary school and higher education. A backwards stepwise regression approach was applied whereby variables with the highest p value were sequentially excluded and model Akaike Information Criterion (AIC) values were compared until a model with the lowest AIC value had been reached. Sex, a non-significant variable, was included to show it did not influence the model. Highly correlated symptoms were grouped, for example, gastrointestinal symptoms included diarrhoea, vomiting and abdominal pain.

Participants reported to be vaccinated before their visit were excluded from all analyses. Analyses were carried out in Stata V.17.^18^

## Results

The study recruited 2963 participants 0–24 years between October 2019 and June 2021, 2542 of whom were aged 0–18 years. Of these, 2540 were COVID-19 vaccine naïve prior to their visit. RocheS and RocheN results were available for 2477 of 2540 (98%) and 2475 of 2540 (97%) participants, respectively. Of those with ethnicity specified, 17% were non-white ethnic groups (online supplemental tables 3 and 4).

The proportion of children aged 0–15 years in each IMD and IDACI quintile was similar (within 1.5%) for the study overall, and within 10% of their local IMD quintile across the majority of regions with the exception of the North West (<1% in least deprived IMD quintile vs 21% in least deprived IDACI quintile) and South East. Nevertheless, >8% of children aged 0–15 years in the North West had individual-level FASiii scores >11 (where 13 is most affluent) (online supplemental table 5). Responses to selected UK Census questions for children aged 0–15 years were generally in agreement with IMD and IDACI quintiles. but did not clearly differentiate between the scoring systems.

In total, 628 of 2477 (25%) were children of healthcare workers.

### Comparison of assays

Of the 2472 participants with results for both assays, 218 (9%) were both positive, and 2215 (90%) both negative with 40 (1%) having discordant results (online supplemental figure 1). The majority (35 of 40, 88%) of discordant results were RocheS positive and RocheN negative. Here we report RocheS results (online supplemental materials include RocheN results).

### Seroprevalence

**Figure 2:**
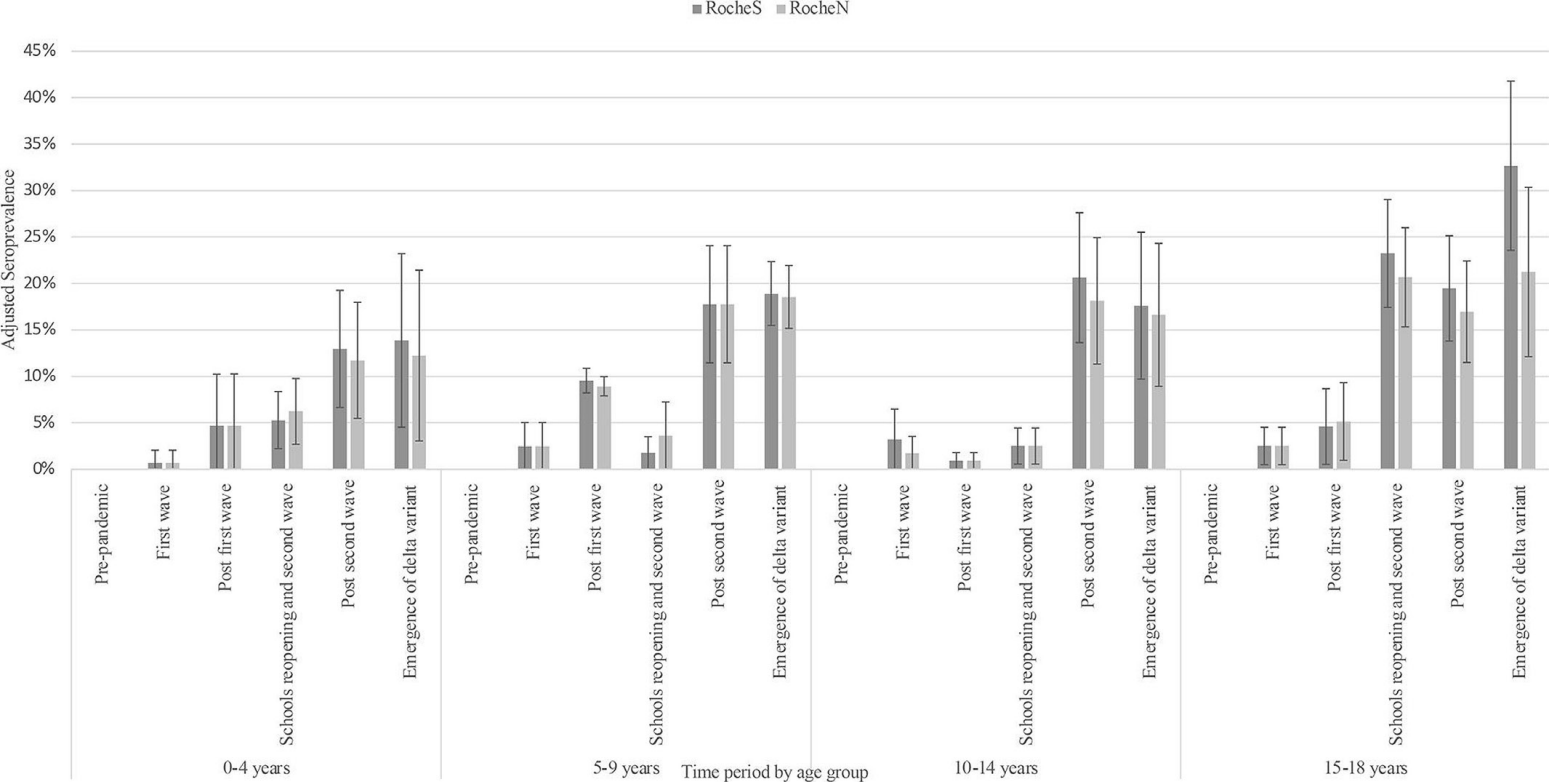
SARS-CoV-2 seroprevalence (RocheS (anti-SARS-CoV-2 IgG spike protein antibodies) and RocheN (anti-SARS-CoV-2 IgG nucleocapsid antibodies)) by age group and time period October 2019–June 2021 in England, adjusted for National Health Service region and ethnicity. Error bars indicate 95% CI.

Overall seroprevalence, adjusted for age, ethnicity and NHS region, increased over time (figure 1 and online supplemental table 6) from 0 in October 2019–March 2020 to 20% (95% CI 16% to 24%) in April 2021–June 2021. For all age groups, seroprevalence remained relatively stable (10% or below) until September–December 2020, where those aged 15–18 years old increased to 23%, followed by an increase in all age groups from January 2021 onwards. By April–June 2021, the adjusted seroprevalence was 20%, varying from 14% in children aged 0–4 years old to 33% in those aged 15–18 years old (figure 2 and online supplemental table 7).

**Figure 3:**
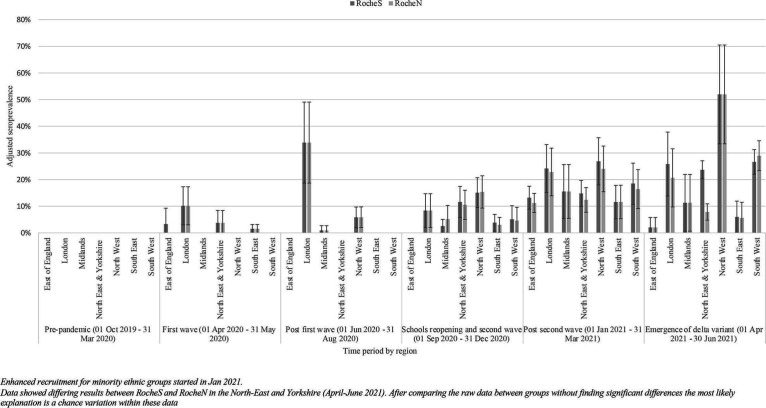
SARS-CoV-2 seroprevalence (RocheS (anti-SARS-CoV-2 IgG spike protein antibodies) and RocheN (anti-SARS-CoV-2 IgG nucleocapsid antibodies)) by region and time period October 2019–June 2021 in England, adjusted for age and ethnicity. Error bars indicate 95% CI.

In June–August 2020, the highest age and ethnicity-adjusted seroprevalence was recorded in London (34%, 95% CI 19% to 49%). In April–June 2021, the highest seroprevalence was in the North West (52%, 95% CI 33% to 71%) (figure 3 and online supplemental table 8).

### Presence of symptoms in seropositive participants

**Figure 4:**
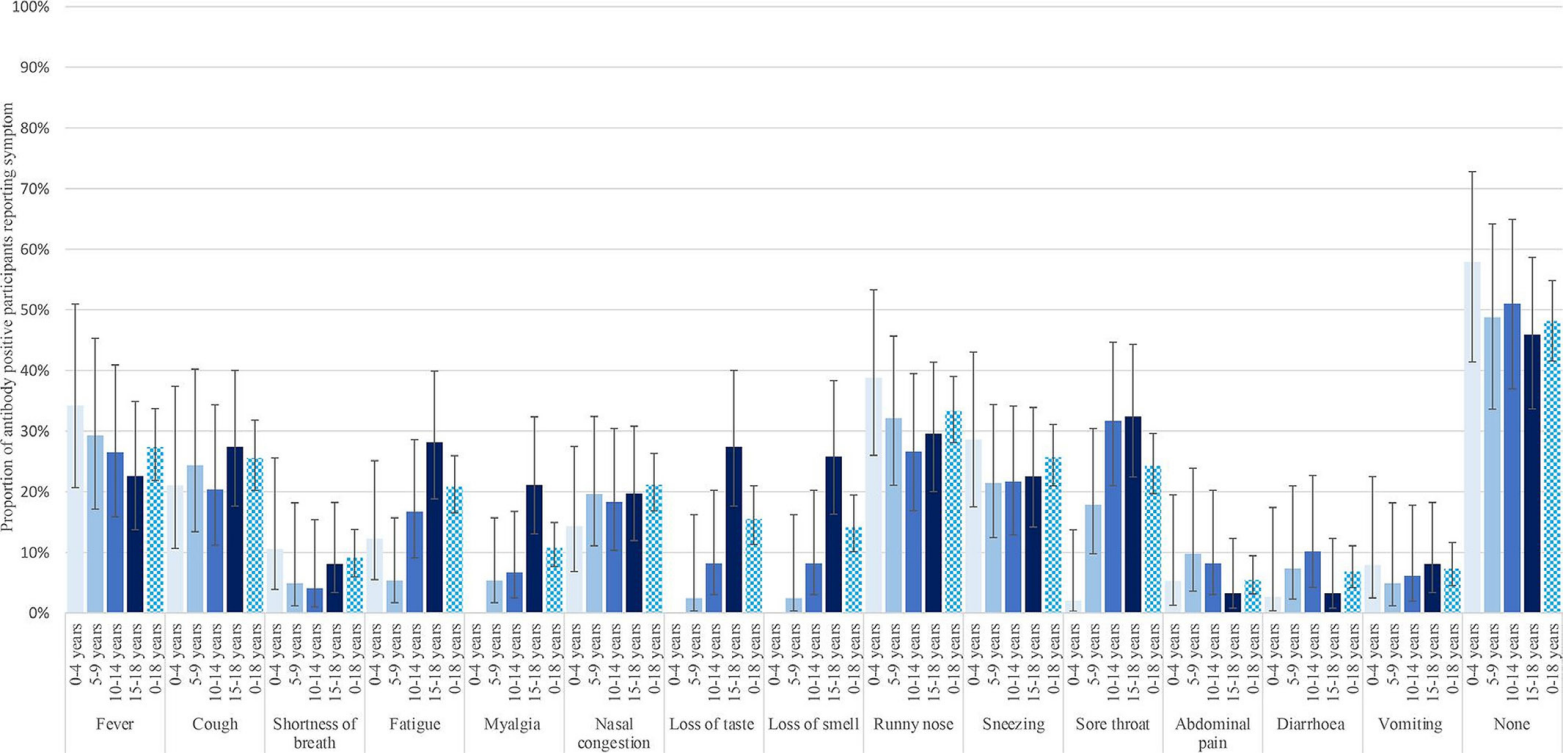
Summary of symptoms reported by participants seropositive on RocheS (anti-SARS-CoV-2 IgG spike protein antibodies) by age group.

Overall, 48% (95% CI 42% to 55%) of seropositive participants reported no symptoms (figure 4 and online supplemental figure 2). Fever was reported in 29% of seropositive participants, and in a univariate analysis was predictive of SARS-CoV-2 positivity in those aged 10–18 years old only (online supplemental figure 3). No solicited symptoms were individually predictive of seropositivity in children aged 0–9 years old.

Backwards stepwise regression demonstrated fever and loss of taste and smell were significant in the older cohort (10–18 years). No symptom clusters were predictive in children 0–9 years old, and fever was of borderline significance in predicting seropositivity (online supplemental table 9).

### Risk factor analysis

The risk of a RocheS seropositive result on a univariate analysis was stable across children 0–14 years old with an increased risk in those 15–18 years old (OR 1.5, 95% CI 1.0 to 2.1, p=0.08), and increase persisting in the multivariable analysis (OR between 1.4 and 1.5, p≤0.05) (table 1 and online supplemental table 10). The North West and London NHS regions showed a higher seropositivity compared with South East (baseline). A higher proportion of children were seropositive living in urban areas than rural areas. Children in minority ethnic groups showed a significantly higher risk in the multivariable analyses compared with their white counterparts (OR 1.4, p=0·04). On univariate analysis, a significant trend towards higher risk of seropositivity in the areas with higher deprivation was seen, however, this was not statistically significant in the multivariable analysis when using either IDACI and IMD. A healthcare worker in the family increased the risk of seropositivity (OR 1.6, 95% CI 1.2 to 2.2, p=0.001).

**Table 1:** Univariate and multivariable logistic regression models to establish risk of SARS-CoV-2 seropositivity on Roche Elecsys Anti-SARS-CoV-2 serological assays for the detection of anti-SARS-CoV-2 IgG spike protein antibodies (RocheS (anti-SARS-CoV-2 IgG spike protein antibodies)) in children aged 0–18 years

Number of participants in model	Univariate		Multivariable					
			IMD deprivation quintiles		IDACI*† deprivation quintiles		IMD and incl HCW†	
			2287		2287		1886	
	OR (95% CI)	LR test (p value)	OR (95% CI)	LR test‡ (p value)	OR (95% CI)	LR test (p value)	OR (95% CI)	LR test (p value)
Age group								
0–4 years	1.1 (0.7 to 1.6)	0.08	0.8 (0.5 to 1.2)	0.01	0.8 (0.5 to 1.2)	0.01	0.8 (0.5 to 1.2)	0.05
5–9 years	1.0 (0.7 to 1.4)		1.0 (0.6 to 1.4)		1.0 (0.6 to 1.4)		0.9 (0.6 to 1.4)	
10–14 years	1 (ref)		1 (ref)		1 (ref)		1 (ref)	
15–18 years	1.5 (1.0 to 2.1)		1.5 (1.1 to 2.2)		1.5 (1.1 to 2.2)		1.4 (1.0 to 2.2)	
Sex								
Female	1 (ref)		1 (ref)		1 (ref)		1 (ref)	
Male	0.9 (0.7 to 1.2)	0.7	1.0 (0.8 to 1.3)	0.9	1.0 (0.8 to 1.3)	1.0	1.0 (0.7 to 1.3)	1.0
NHS region								
East of England	1.5 (0.7 to 3.0)	<0.001	1.1 (0.5 to 2.4)	<0.001	1.1 (0.5 to 2.3)	<0.001	1.0 (0.4 to 2.3)	<0.001
London	6.0 (3.7 to 9.5)		3.1 (1.9 to 5.2)		3.1 (1.8 to 5.2)		3.0 (1.7 to 5.3)	
Midlands	1.7 (0.9 to 3.2)		1.2 (0.6 to 2.5)		1.2 (0.6 to 2.4)		1.1 (0.5 to 2.4)	
North East and Yorkshire	2.7 (1.6 to 4.3)		1.9 (1.1 to 3.2)		1.8 (1.1 to 3.1)		1.8 (1.0 to 3.2)	
North West	4.7 (2.9 to 7.5)		2.9 (1.7 to 5.2)		2.8 (1.6 to 4.9)		2.8 (1.6 to 5.0)	
South East	1 (ref)		1 (ref)		1 (ref)		1 (ref)	
South West	1.8 (1.0 to 3.0)		1.6 (0.9 to 2.8)		1.6 (0.9 to 2.8)		1.6 (0.9 to 2.9)	
Time period								
Pre-pandemic (01 Oct 2019–31 Mar 2020)		<0.001		<0.001		<0.001		<0.001
First wave (01 Apr 2020–31 May 2020)	0.2 (0.1 to 0.3)		0.2 (0.1 to 0.4)		0.2 (0.1 to 0.4)			
Post-first wave (01 Jun 2020–31 Aug 2020)	0.2 (0.1 to 0.3)		0.2 (0.1 to 0.3)		0.2 (0.1 to 0.3)		0.2 (0.1 to 0.3)	
Schools reopening and second wave (01 Sep 2020–31 Dec 2020)	0.4 (0.2 to 0.5)		0.4 (0.2 to 0.6)		0.4 (0.3 to 0.6)		0.4 (0.3 to 0.6)	
Post-second wave (01 Jan 2021–31 Mar 2021)	1 (ref)		1 (ref)		1 (ref)		1 (ref)	
Emergence of delta variant (01 Apr 2021–30 Jun 2021)	1.2 (0.8 to 1.7)		1.0 (0.7 to 1.5)		1.0 (0.7 to 1.6)		1.0 (0.7 to 1.6)	
Ethnicity								
White	1 (ref)	<0.001	1 (ref)		1 (ref)		1 (ref)	
Minority ethnic group§	2.5 (1.8 to 3.3)		1.4 (1.0 to 2.0)	0.04	1.4 (1.0 to 2.0)	0.04	1.4 (1.0 to 2.0)	0.07
IMD deprivation quintile								
Most deprived 1	2.5 (1.7 to 3.7)	<0.001	1.4 (0.8 to 2.2)	0.1			1.6 (0.9 to 2.6)	0.04
2	1.6 (1.0 to 2.5)		0.8 (0.5 to 1.2)				0.7 (0.4 to 1.2)	
3	1.1 (1.0 to 2.3)		1.0 (0.6 to 1.5)				1.1 (0.7 to 1.7)	
4	1.5 (1.0 to 2.2)		1.2 (0.8 to 1.9)				1.3 (0.8 to 2.0)	
Least deprived 5	1 (ref)		1 (ref)				1 (ref)	
IDACI deprivation quintile								
Most deprived 1	1.9 (1.3 to 2.8)	<0.001			1.2 (0.8 to 1.9)	0.1		
2	1.1 (0.7 to 1.6)				0.6 (0.4 to 1.0)			
3	1.2 (0.8 to 1.7)				1.0 (0.6 to 1.5)			
4	1.0 (0.7 to 1.5)				0.9 (0.6 to 1.4)			
Least deprived 5	1 (ref)				1 (ref)			
Urban/rural								
Rural	0.3 (0.2 to 0.5)	<0.001	0.6 (0.3 to 1.0)	0.03	0.6 (0.3 to 1.0)	0.03	0.6 (0.4 to 1.1)	0.07
Urban	1 (ref)		1 (ref)		1 (ref)		1 (ref)	
HCW*								
No	1 (ref)	0.002					1 (ref)	
Yes	1.5 (1.2 to 2.0)						1.6 (1.2 to 2.2)	0.001

Multivariable analysis adjusts for age, sex, NHS region, time period, ethnicity, socioeconomic deprivation (using either IMD or IDACI), urban/rural geography and presence of HCWs within the household.

*Fewer results available for this analysis due to incomplete data in HCW field (see online supplemental table 3).

†IDACI measures the proportion of all children aged 0–15 years living in income-deprived families.

‡LR for each risk factor calculated with overall p value displayed.

§Minority ethnic group includes all minority groups apart from white minorities.

HCW, healthcare worker; IDACI, Income Deprivation Affecting Children Index; IMD, Index of Multiple Deprivation; LR, likelihood ratio; NHS, National Health Service.

## Discussion

Here we report results of a community-based study recruiting a representative cohort of the paediatric population in England from the start of the SARS-CoV-2 pandemic, with extensive characterisation of demography and potential COVID-19 symptoms. This provides a unique assessment of the prevalence and risk factors for naturally acquired antibodies against the SARS-CoV-2 virus in children and adolescents prior to introduction of widescale immunisation. Notably, approximately one-third of participants aged 15–18 years old had antibodies against SARS-CoV-2 in April–June 2021, prior to immunisation or the further wave of infections in this age group that occurred in Autumn 2021.

This study adds to the evidence base regarding paediatric SARS-CoV-2 seroprevalence, building on other studies in England and/or the UK, including SKIDS^4 5^ (recruiting from educational settings) and COVID Warriors^3^ (recruiting children of healthcare workers). Together these seroprevalence studies provide estimates of the levels of seropositivity within the paediatric population, vital for modelling of disease susceptibility and immunisation planning, complementing insights gained from repeat cross-sectional community infection surveys such as REACT-1^2^ and the COVID-19 infection survey.^19^

When comparing studies at similar time points, differences in estimates emerge. In April–August 2020, overall rates of seropositivity in our data were approximately half those of COVID Warriors,^3^ likely due to the elevated risk of seropositivity in household members of healthcare workers. In June 2021, our age-adjusted estimates were higher than the SKIDS Study, which reports 11.25% and 12.95% of primary and secondary school pupils being seropositive using oral fluid sampling,^20^ compared with 18.9% and 32.7% in similar age groups in our study. This may reflect differences in sensitivity in antibody detection in saliva (75%) vs serum (95.5%).^14 21^ Lastly, ONS (Office for National Statistics) data in summer 2021 showed a seroprevalence of 47% in participants aged 16–24 years old^19^ compared with 32.7% in those aged 15–18 years old in our study, a variance potentially accounted for by higher infection rates observed in those aged 18–24 years old in the period,^22^ along with the possibility of vaccine-induced immunity in this older age group.

More consistency is seen when comparing trends over time. The dramatic increase in seropositivity evident in September–December 2020 in participants 15–18 years old, followed by all younger age groups (including those 0–4 years old) in January 2021–March 2021 is consistent with antibody data from SKIDS showing an increase in infection rates in those aged 11–18 years old in September–December 2020.^5^ A further increase in seropositivity in participants aged 15–18 years old in April–June 2021 corresponds to the emergence of the delta variant, and associated increase in adolescent (13–17 years old) infections demonstrated in REACT-1 antigen data in May–July 2021.^2^ COVID-19 seroprevalence varied across regions, with an early (June–August 2020) increase in London, again consistent with COVID Warriors and REACT Studies,^3^ while the striking increase in seropositivity rates in the North West from May to June 2021 is reflected in regional data from the COVID-19 infection survey.

Our data showed a greater than twofold increased risk for participants from a minority ethnic group compared with white participants, which remained elevated in the multivariable analysis (including adjustment for socioeconomic status). This is consistent with the increased risk of SARS-CoV-2 infection from minority ethnic groups reported in the adult and paediatric populations since the first peak of the pandemic in England. Ward *et al* showed a threefold increase in risk of being antibody positive in the adult black population in England.^1^ This trend was also reported by Ladhani *et al* in primary school children in June 2020.^23^ Our data were not powered to look at individual ethnic groups, nevertheless we show that belonging to a minority ethnic group is a significant risk factor in the paediatric population (independent of socioeconomic status and adult-type comorbidities). Of note is that this increased risk became of borderline significance when the presence of a healthcare worker in the household was included in the multivariable analysis, although this also may reflect the limitations of a smaller dataset. Nevertheless, the increased susceptibility among minority ethnic groups requires further study as to whether it reflects reduced access to healthcare or other social equalities.

Unlike COVID Warriors, we did not demonstrate that gastrointestinal symptoms were predictive of seropositivity.^3^ This may reflect a symptomatology specific to ancestral lineages A and B which were in circulation when COVID Warriors collected their initial data,^16^ or differing methods of data collection. Of note is that in our study, symptomatology questionnaires were adapted as the study progressed, with some symptoms collected retrospectively creating the potential for recall bias. Furthermore, while the age discrepancy in reporting of anosmia and ageusia is striking, this may reflect difficulties in children <10 years of age communicating these symptoms.

The strengths of this study are that it includes participants aged 0–18 years old and has sampled from all NHS regions in England which allows generalisations to be made to England as a whole. To our knowledge, at the time of publication, this is the only paediatric study in England that has a community-based recruitment strategy with efforts to recruit a representative sample of the region. The study has several limitations, including having been adapted from a pre-pandemic design. One-quarter of participants were children of healthcare workers, which is higher than the general population.^24^ Both the RocheS and RocheN assays have primarily been validated in the adult, rather than paediatric population, and there is the possibility that the SARS-CoV-2 nucleocapsid-specific IgG response to infection, and the longevity of that response, may differ in younger age groups.^25^ Of note is the increased divergence between RocheS and RocheN assays in later time periods. As participants with known COVID-19 vaccinations (which would lead to a positive RocheS while RocheN remained negative) were excluded from the analysis, and widespread immunisation was not conducted in the relevant age groups during the period studied, this may reflect more rapid waning of RocheN than RocheS, which has been seen in the adult literature.^26^

This study has provided a gold-standard SARS-CoV-2 seroprevalence dataset as part of a unique biobank of serum samples from children and young adults aged 0–24 years with a comprehensive history of immunisation and demography. The willingness of participants and their families to participate in research has created an invaluable resource for understanding COVID-19 and other infectious diseases.

## Data Availability

Data are available upon reasonable request. After publication, anonymised individual patient data will be made available upon request to the corresponding author for secondary research, conditional on assurance from the secondary researcher that the proposed use of the data is compliant with the Medical Research Council Policy on Data Sharing regarding scientific quality, ethical requirements, and value for money, and is compliant with the National Institute for Health Research policy on data sharing. A minimum requirement with respect to scientific quality will be a publicly available prespecified protocol describing the purpose, methods, and analysis of the secondary research (eg, a protocol for a Cochrane systematic review), approved by a UK Research Ethics Committee or other similar, approved ethics review body. Participant identifiers will not be passed on to any third party. Data will be available for 5 years after publication.
