# Supplementary material for: Community seroprevalence of SARS-CoV-2 in children and adolescents in England, 2019–2021
Source: Arch Dis Child. 2022 Jul 20;108(2):123–30. doi: 10.1136/archdischild-2022-324375 (PMC9887370; doi:10.1136/archdischild-2022-324375)
Supplement: Supplementary data [file archdischild-2022-324375supp001.pdf]

Table 1 Recruiting sites NHS region(s) and postcode districts where applicable

Site	NHS region(s)	Date of first recruit	Selected postcode districts						
**Bradford**	Yorkshire and Humber	31/3/2020	BD15	BD6					
**Bristol**	South West	9/4/2020	BS2	BS20	BS3	BS37	BS41	BS8	
**Imperial**	London	8/2/2021	SW1	SW3	NW6	NW8	W11	WC2	
**Leeds**	Yorkshire and Humber	5/6/2020	LS25	WF2					
**Manchester**	North West	12/6/2020	M1	M12	M20	M23	M25	M6	
**Newcastle**	North East	7/1/2021	NE3	NE12	NE13	NE20	NE23	NE28	
**Nottingham***	Midlands	10/7/2020							
**Oxford**	South East<br>East of England	15/10/2019	HP17	HP22	HP23	MK13	OX1	OX11	OX28
**Plymouth***	South West	3/6/2020							
**Sheffield**	Yorkshire and Humber Midlands	20/4/2020	S14	S43					
**Southampton**	South East	12/4/2020	SO16	SO23	SO24	SO50	SO51	SO52	
**St Georges London**	London<br>South East	26/3/2020	CR4	KT18	KT6	SM3	SW11	SW20	
**West Suffolk**	East of England	21/1/2021	CB8	CB9	CO10	IP14	IP24	IP28	

**recruitment not restricted by postcode district*

Table 2 Sensitivity, specificity and thresholds of positivity for anti-SARS-CoV-2 IgG spike protein antibodies (RocheS) and anti-SARS-CoV-2 IgG nucleocapsid antibodies (RocheN) [13,14]

	RocheS	RocheN
**Threshold of positivity**	0·8U/ml	1·0 COI
**Sensitivity**	95·5% (95%CI 93·2-97·1)	83·9% (95% CI 74·8-90·7)
**Specificity**	100% (95% CI 99·1-100)	100% (95% CI 99·1-100)

COI – cut off index
U/ml - *units per millilitre*

Table 3 Summary of participants aged 0-18 years tested for COVID-19 with RocheS - Anti-SARS-CoV-2 IgG spike protein antibodies and RocheN - Anti-SARS-CoV-2 IgG nucleocapsid antibodies assays

	Seroprevalence		Symptoms		Risk factor	
	RocheS	RocheN	RocheS	RocheN	RocheS	RocheN
**Age group**						
**0-4 years**	532 (21·5%)	529 (21·4%)	289 (21·6%)	287 (21·5%)	516 (21·4%)	513 (21·3%)
**5-9 years**	636 (25·7%)	636 (25·7%)	344 (25·7%)	344 (25·7%)	620 (25·7%)	620 (25·7%)
**10-14 years**	721 (29·1%)	721 (29·1%)	377 (28·2%)	377 (28·2%)	705 (29·2%)	705 (29·3%)
**15-18 years**	588 (23·7%)	589 (23·8%)	329 (24·6%)	329 (24·6%)	571 (23·7%)	572 (23·7%)
**Sex**						
**Female**	1230 (49·7%)	1230 (49·7%)	689 (51·5%)	688 (51·5%)	1198 (49·7%)	1198 (49·7%)
**Male**	1247 (50·3%)	1245 (50·3%)	650 (48·7%)	649 (48·5%)	1214 (50·3%)	1212 (50·3%)
**NHS region**						
**East of England**	171 (6·9%)	171 (6·9%)	96 (7·2%)	96 (7·2%)	156 (6·5%)	156 (6·5%)
**London**	289 (11·7%)	289 (11·7%)	181 (13·6%)	181 (13·5%)	287 (11·9%)	287 (11·9%)
**Midlands**	210 (8·5%)	211 (8·5%)	138 (10·3%)	138 (10·3%)	207 (8·6%)	208 (8·6%)

**North East and Yorkshire**	419 (16·9%)	419 (16·9%)	232 (17·3%)	232 (17·4%)	410 (17·0%)	410 (17·0%)
**North West**	331 (13·4%)	331 (13·4%)	141 (10·5%)	141 (10·5%)	326 (13·5%)	326 (13·5%)
**South East**	633 (25·6%)	631 (25·5%)	260 (19·4%)	258 (19·3%)	607 (25·2%)	605 (25·1%)
**South West**	424 (17·1%)	423 (17·1%)	291 (21·7%)	291 (21·8%)	419 (17·4%)	418 (17·3%)
**Time period**						
**Pre-pandemic (01 Oct 2019 - 31 Mar 2020)**	125 (5·0%)	125 (5·1%)	32 (2·4%)	32 (2·4%)	125 (5·2%)	125 (5·2%)
**First wave (01 Apr 2020 - 31 May 2020)**	335 (13·5%)	335 (13·5%)	74 (5·5%)	74 (5·5%)	334 (13·8%)	334 (13·9%)
**Post first wave (01 Jun 2020 - 31 Aug 2020)**	516 (20·8%)	515 (20·8%)	111 (8·3%)	111 (8·3%)	507 (21·0%)	506 (21·0%)
**Schools reopening and second wave (01 Sep 2020 - 31 Dec 2020)**	464 (18·7%)	464 (18·7%)	298 (22·3%)	297 (22·2%)	456 (18·9%)	456 (18·9%)
**Post second wave (01 Jan 2021 - 31 Mar 2021)**	771 (31·1%)	770 (31·1%)	617 (46·1%)	616 (46·1%)	736 (30·5%)	735 (30·5%)
**Emergence of delta variant (01 Apr 2021 - 30 Jun 2021)**	266 (10·7%)	266 (10·7%)	207 (15·5%)	207 (15·5%)	254 (10·5%)	254 (10·5%)
**Ethnicity**						
**White (White and White minorities)**	1991 (80·4%)	1989 (80·4%)	1073 (80·1%)	1071 (80·1%)	1987 (82·4%)	1985 (82·4%)
**Black (African, Caribbean, Other Black background)**	51 (2·1%)	51 (2·1%)	29 (2·2%)	29 (2·2%)	51 (2·1%)	51 (2·1%)
**Asian (Bangladeshi, Pakistani, Indian, Chinese, Arab, other Asian background and other ethnic group)**	144 (5·8%)	144 (5·8%)	99 (7·4%)	99 (7·4%)	144 (6·0%)	144 (6·0%)
**Multiple ethnic backgrounds**	231 (9·3%)	231 (9·3%)	134 (10·0%)	134 (10·0%)	230 (9·5%)	230 (9·5%)
**Not specified**	60 (2·4%)	60 (2·4%)	4 (0·3%)	4 (0·3%)		

**IMD quintile**						
**Most deprived 1**	357 (14·4%)	357 (14·4%)	179 (13·4%)	179 (13·4%)	352 (14·6%)	352 (14·6%)
**2**	337 (13·6%)	336 (13·6%)	178 (13·3%)	177 (13·2%)	328 (13·6%)	327 (13·6%)
**3**	461 (18·6%)	461 (18·6%)	247 (18·4%)	247 (18·5%)	446 (18·5%)	446 (18·5%)
**4**	535 (21·7%)	534 (21·6%)	289 (21·6%)	289 (21·6%)	518 (21·5%)	517 (21·5%)
**Least deprived 5**	782 (31·6%)	782 (31·6%)	441 (33·0%)	440 (32·9%)	768 (31·8%)	768 (31·9%)
**Not specified**	5 (0·2%)	5 (0·2%)	5 (0·4%)	5 (0·4%)		
**IDACI quintile**						
**Most deprived 1**	374 (15·1%)	374 (15·1%)	188 (14·0%)	188 (14·1%)	368 (15·3%)	368 (15·3%)
**2**	348 (14·0%)	347 (14·0%)	194 (14·5%)	193 (14·4%)	342 (14·2%)	341 (14·1%)
**3**	451 (18·2%)	451 (18·2%)	225 (16·8%)	225 (16·8%)	433 (18·0%)	433 (18·0%)
**4**	549 (22·2%)	541 (21·9%)	305 (22·8%)	305 (22·8%)	538 (22·3%)	537 (22·3%)
**Least deprived 5**	750 (30·3%)	750 (30·3%)	422 (31·5%)	421 (31·5%)	731 (30·3%)	731 (30·3%)
**Not specified**	5 (0·2%)	5 (0·2%)	5 (0·4%)	5 (0·4%)		
**Urban/rural**						
**Urban**	1984 (80·1%)	1982 (80·1%)	1076 (80·4%)	1075 (80·4%)	1933 (80·1%)	1931 (80·1%)
**Rural**	493 (19·9%)	493 (19·9%)	263 (19·6%)	262 (19·6%)	479 (19·9%)	479 (19·9%)
**Member of family is a healthcare worker**						
**Yes**	628 (25·4%)	627 (25·3%)	421 (31·5%)	421 (31·5%)	626 (26·0%)	625 (25·9%)
**No**	1269 (51·2%)	1268 (51·2%)	804 (60·0%)	802 (60·0%)	1262 (52·3%)	1261 (52·3%)
**Not specified**	580 (23·4%)	580 (23·4%)	114 (8·5%)	114 (8·5%)	524 (21·7%)	524 (21·7%)
**Total**	2477	2475	1339	1337	2412	2410

Table 4 Comparing regional populations (0-18 years) with the STORY sample (0-18 years olds) including vaccinated individuals

**NHS region**	**East of England**		**London**		**Midlands**		**North East and Yorkshire**		**North West**		**South East**		**South West**	
	**Region (0-18 years (%))**	**STORY sample (0-18 years (%))**	**Region (0-18 years (%))**	**STORY sample (0-18 years (%))**	**Region (0-18 years (%))**	**STORY sample (0-18 years (%))**	**Region (0-18 years (%))**	**STORY sample (0-18 years (%))**	**Region (0-18 years (%))**	**STORY sample (0-18 years (%))**	**Region (0-18 years (%))**	**STORY sample (0-18 years (%))**	**Region (0-18 years (%))**	**STORY sample (0-18 years (%))**
**White***	1146365·8 (82·4)	140 (88·6)	998497·8 (45·4)	199 (67·7)	852484·5 (75·4)	178 (84·8)	941658·8 (89·5)	361 (86·8)	1309834·6 (80·8)	235 (68·5)	1709830·1 (82·5)	531 (84·4)	1045274·7 (90·7)	393 (91·2)
**Black****	46559·0 (3·3)	2 (1·3)	374912·9 (17·0)	19 (6·5)	37043·3 (3·3)	4 (1·9)	19364·3 (1·8)	7 (1·7)	36393·4 (2·2)	14 (4·1)	55537·4 (2·7)	4 (0·6)	18522·2 (1·6)	2 (0·5)
**Asian*****	111665·8 (8·0)	4 (2·5)	518125·2 (23·5)	30 (10·2)	169453·2 (15·0)	12 (5·7)	32584·5 (3·1)	17 (4·0)	190051·5 (11·7)	49 (14·3)	174174·6 (8·4)	28 (4·4)	36856·3 (3·2)	10 (2·3)
**Multiple ethnic backgrounds**	86007·6 (6·2)	12 (7·6)	310162·9 (14·1)	46 (15·6)	71616·5 (6·3)	16 (7·6)	58044·7 (5·5)	31 (7·5)	84851·4 (5·2)	45 (13·1)	131887·0 (6·4)	67 (10·6)	51275·7 (4·5)	26 (6·0)
**total**	1390598·2	158·0	2201698·7	294·0	1130597·5	210·0	1051652·4	416·0	1621130·9	343·0	2071429·2	630·0	1151928·9	431·0

*White including white minorities
**Black (African, Caribbean, Other Black background)
*** Asian (Bangladeshi, Chinese, Indian, Pakistani, Other Asian background)

Ratcliffe H, *et al. Arch Dis Child* 2023; 108:123–130. doi: 10.1136/archdischild-2022-324375

Table 5 Comparing children aged 0-15 years on IDACI and IMD by region as a measure of socioeconomic deprivation in children IMD and IDACI derived from postcode of participant, FASiii and Census 2011 home ownership and house size questions data were collected on the questionnaire

		Quintile				
		1	2	3	4	5
**Overall**	**IMD**	303	280	409	453	663
	**Number (%) of families that own their own home in each IMD quintile**	50·8	69·4	77·9	81·7	85·8
	**Average number of rooms in home in each IMD quintile**	5·6	6·1	6·6	7·0	7·3
	**Average Number of bedrooms in home in each IMD quintile**	2·9	3·2	3·5	3·6	3·8
	**Mean FASiii score in each IMD quintile**	6·5	7·7	8·7	8·9	9·5
	**IDACI**	315	304	481	476	632
	**Number (%) of families that own their own home in each IDACI quintile**	49·8	66·4	79·8	81·6	87·7
	**Average number of rooms in home in each IDACI quintile**	5·6	6·1	6·6	6·8	7·5
	**Average Number of bedrooms in home in each IDACI quintile**	2·9	3·2	3·5	3·6	3·8
	**Mean FASiii score in each IDACI quintile**	6·6	7·7	8·6	8·9	9·5
**East of England**	**IMD**	16	22	44	40	25
	**Number (%) of families that own their own home in each IMD quintile**	30·8	59·1	65·8	86·1	100·0
	**Average number of rooms in home in each IMD quintile**	5·8	6·6	6·8	7·4	7·8
	**Average Number of bedrooms in home in each IMD quintile**	2·9	3·5	3·3	3·9	3·7
	**Mean FASiii score in each IMD quintile**	6·4	7·6	7·8	9·3	9·7
	**IDACI**	24	22	30	40	31
	**Number (%) of families that own their own home in each IDACI quintile**	33·3	59·1	72·0	88·6	93·1
	**Average number of rooms in home in each IDACI quintile**	6·2	5·2	7·2	7·1	8·0
	**Average Number of bedrooms in home in each IDACI quintile**	3·0	3·4	3·8	3·5	3·8
	**Mean FASiii score in each IDACI quintile**	6·5	7·2	9·2	8·5	9·7
**London**	**IMD**	9	41	61	48	79
	**Number (%) of families that own their own home in each IMD quintile**	22·2	36·6	73·8	83·3	84·6
	**Average number of rooms in home in each IMD quintile**	4·8	5·1	5·9	6·7	6·8
	**Average Number of bedrooms in home in each IMD quintile**	2·3	2·9	3·2	3·5	3·8
	**Mean FASiii score in each IMD quintile**	5·8	7·5	8·5	9·3	9·7
	**IDACI**	18	55	35	61	69
	**Number (%) of families that own their own home in each IDACI quintile**	27·8	50·9	74·3	80·3	88·2
	**Average number of rooms in home in each IDACI quintile**	4·6	5·3	6·1	6·4	7·0
	**Average Number of bedrooms in home in each IDACI quintile**	2·5	2·9	3·3	3·6	3·9
	**Mean FASiii score in each IDACI quintile**	7·2	7·5	8·3	9·2	10·1
**Midlands**	**IMD**	17	30	34	30	44
	**Number (%) of families that own their own home in each IMD quintile**	58·8	67·9	84·8	86·7	95·3
	**Average number of rooms in home in each IMD quintile**	6·8	6·3	6·4	7·1	7·8
	**Average Number of bedrooms in home in each IMD quintile**	3·6	3·4	3·5	3·7	4·0
	**Mean FASiii score in each IMD quintile**	7·5	8·3	8·8	9·1	9·8
	**IDACI**	23	32	31	35	34
	**Number (%) of families that own their own home in each IDACI quintile**	52·2	75·9	83·9	91·2	97·1
	**Average number of rooms in home in each IDACI quintile**	6·6	6·4	6·3	7·3	7·9
	**Average Number of bedrooms in home in each IDACI quintile**	3·4	3·5	3·4	3·9	4·0
	**Mean FASiii score in each IDACI quintile**	7·7	8·5	8·7	9·5	9·7
**North East and Yorkshire**	**IMD**	88	59	61	57	89
	**Number (%) of families that own their own home in each IMD quintile**	58·8	82·5	81·7	91·1	88·6
	**Average number of rooms in home in each IMD quintile**	5·5	6·3	6·8	7·1	7·4

	**Average Number of bedrooms in home in each IMD quintile**	2·9	3·2	3·6	3·6	3·7
	**Mean FASiii score in each IMD quintile**	6·8	7·2	8·7	9·3	9·0
	**IDACI**	83	57	68	41	105
	**Number (%) of families that own their own home in each IDACI quintile**	60·0	73·2	83·3	87·5	92·3
	**Average number of rooms in home in each IDACI quintile**	5·6	6·3	6·8	6·7	7·4
	**Average Number of bedrooms in home in each IDACI quintile**	3·0	3·2	3·5	3·5	3·7
	**Mean FASiii score in each IDACI quintile**	6·7	7·3	8·6	8·8	9·1
**North West**	**IMD**	119	54	71	51	2
	**Number (%) of families that own their own home in each IMD quintile**	51·7	86·5	81·7	85·7	50·0
	**Average number of rooms in home in each IMD quintile**	5·7	6·5	7·3	7·8	8·0
	**Average Number of bedrooms in home in each IMD quintile**	2·9	3·4	3·9	4·2	4·5
	**Mean FASiii score in each IMD quintile**	6·5	8·4	9·0	8·7	9·5
	**IDACI**	116	27	50	41	63
	**Number (%) of families that own their own home in each IDACI quintile**	52·2	74·1	87·5	70·0	91·9
	**Average number of rooms in home in each IDACI quintile**	5·7	6·0	7·3	6·4	8·1
	**Average Number of bedrooms in home in each IDACI quintile**	3·0	3·2	3·7	3·7	4·3
	**Mean FASiii score in each IDACI quintile**	6·5	8·0	8·3	8·5	9·3
**South East**	**IMD**	23	40	81	112	293
	**Number (%) of families that own their own home in each IMD quintile**	27·3	63·2	73·7	76·9	80·4
	**Average number of rooms in home in each IMD quintile**	5·6	5·9	6·1	7·1	7·3
	**Average Number of bedrooms in home in each IMD quintile**	2·7	3·1	3·3	3·5	3·9
	**Mean FASiii score in each IMD quintile**	6·3	7·6	8·4	9·4	9·4
	**IDACI**	19	68	104	152	206
	**Number (%) of families that own their own home in each IDACI quintile**	23·5	65·7	72·6	77·7	82·5
	**Average number of rooms in home in each IDACI quintile**	5·3	6·1	6·5	7·0	7·5
	**Average Number of bedrooms in home in each IDACI quintile**	2·5	3·2	3·5	3·6	3·8
	**Mean FASiii score in each IDACI quintile**	5·5	8·0	8·7	9·2	9·6
**South West**	**IMD**	31	34	57	115	131
	**Number (%) of families that own their own home in each IMD quintile**	54·8	75·8	83·6	76·1	91·3
	**Average number of rooms in home in each IMD quintile**	5·1	5·8	6·9	6·4	7·3
	**Average Number of bedrooms in home in each IMD quintile**	2·8	3·4	3·5	3·5	3·8
	**Mean FASiii score in each IMD quintile**	6·1	7·5	8·5	8·2	9·5
	**IDACI**	32	43	63	106	124
	**Number (%) of families that own their own home in each IDACI quintile**	51·6	71·4	85·2	84·8	85·8
	**Average number of rooms in home in each IDACI quintile**	5·2	6·5	6·4	6·7	7·2
	**Average Number of bedrooms in home in each IDACI quintile**	2·9	3·3	3·3	3·7	3·7
	**Mean FASiii score in each IDACI quintile**	6·3	7·6	8·5	8·6	9·2

Ratcliffe H, *et al. Arch Dis Child* 2023; 108:123–130. doi: 10.1136/archdischild-2022-324375

Figure 1 A comparison of results by the RocheN - Anti-SARS-CoV-2 IgG nucleocapsid antibodies assay and RocheS - Anti-SARS-CoV-2 IgG spike protein antibodies assay by age

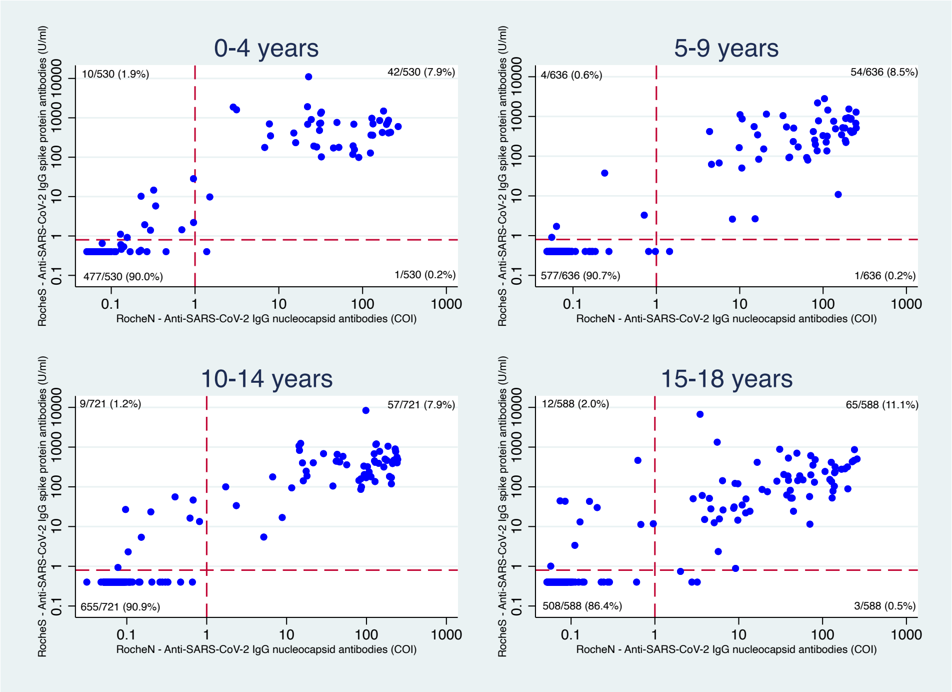

Assay positivity thresholds indicated as red dashed lines (0.8 U/ml for Spike and 1 COI for Nucleocapsid)
COI – cut off index, U/ml (units per millilitre)

Table 6 SARS-CoV-2 seroprevalence (RocheS - Anti-SARS-CoV-2 IgG spike protein antibodies and RocheN - Anti-SARS-CoV-2 IgG nucleocapsid antibodies) by age group and time period October 2019 to June 2021 in England, adjusted for age, region and ethnicity

	**RocheS**			**RocheN**		
**Period**	**N**	**Crude rate**	**Adjusted rate**	**N**	**Crude rate**	**Adjusted rate**
**Pre-pandemic (Oct 19 - Mar 20)**	125	0·0%	0·0%	125	0·0%	0·0%
**First wave (Apr 20 - May 20)**	335	3·9%	2·2% (0·9%-3·4%)	335	3·6%	1·8% (0·8%-2·8%)
**Post first wave (Jun 20 - Aug 20)**	516	3·9%	5·2% (3·2%-7·2%)	515	3·7%	5·1% (3·1%-7·2%)
**Schools reopening and second wave (Sep 20 - Dec 20)**	464	6·9%	7·1% (5·5%-8·6%)	464	6·0%	7·3% (5·5%-9·1%)
**Post second wave (Jan 21 - Mar 21)**	771	17·5%	17·6% (14·4%-20·9%)	770	15·2%	16·2% (12·9%-19·4%)
**Emergence of delta variant (Apr 21 - Jun 21)**	266	19·9%	19·9% (16·1%-23·7%)	266	17·7%	16·8% (13·1%-20·6%)

Table 7 SARS-CoV-2 seroprevalence (RocheS - Anti-SARS-CoV-2 IgG spike protein antibodies and RocheN - Anti-SARS-CoV-2 IgG nucleocapsid antibodies) by age group and time period October 2019 to June 2021 in England, adjusted for NHS region and ethnicity

		RocheS			RocheN		
		**N**	**Crude**	**Adjusted**	**N**	**Crude**	**Adjusted**
**0-4 years**	**Pre-pandemic (Oct 19 - Mar 20)**	25	0·0%	0·0%	25	0·0%	0·0%
	**First wave (Apr 20-May 20)**	62	1·6%	0·7% (0·0%-2·1%)	62	1·6%	0·7% (0·0%-2·1%)
	**Post first wave (Jun 20 - Aug 20)**	64	4·7%	4·7% (0·0%-10·2%)	63	4·8%	4·7% (0·0%-10·3%)
	**Schools reopening and second wave (Sep 20 - Dec 20)**	77	5·2%	5·3% (2·2%-8·4%)	76	6·6%	6·2% (2·7%-9·8%)
	**Post second wave (Jan 21 -Mar 21)**	200	13·5%	13·0% (6·7%-19·3%)	199	10·1%	11·7% (5·5%-18·0%)
	**Emergence of delta variant (Apr 21 - Jun 21)**	104	16·3%	13·9% (4·5%-23·2%)	104	13·5%	12·2% (3·1%-21·4%)
**5-9 years**	**Pre-pandemic (Oct 19 - Mar 20)**	31	0·0%	0·0%	31	0·0%	0·0%
	**First wave (Apr 20-May 20)**	83	3·6%	2·5% (0·0%-5·0%)	83	3·6%	2·5% (0·0%-5·0%)
	**Post first wave (Jun 20 - Aug 20)**	144	4·9%	9·5% (8·2%-10·9%)	144	3·5%	8·9% (7·9%-10·0%)
	**Schools reopening and second wave (Sep 20 - Dec 20)**	119	3·4%	1·8% (0·0%-3·5%)	119	3·4%	3·6% (0·0%-7·3%)
	**Post second wave (Jan 21 -Mar 21)**	187	15·0%	17·8% (11·5%-24·1%)	187	15·0%	17·8% (11·5%-24·1%)
	**Emergence of delta variant (Apr 21 - Jun 21)**	72	22·2%	18·9% (15·5%-22·3%)	72	20·8%	18·5% (15·2%-21·9%)
**10-14 years**	**Pre-pandemic (Oct 19 - Mar 20)**	41	0·0%	0·0%	41	0·0%	0·0%
	**First wave (Apr 20-May 20)**	108	3·7%	3·2% (0·0%-6·5%)	108	2·8%	1·7% (0·0%-3·5%)
	**Post first wave (Jun 20 - Aug 20)**	174	2·3%	0·9% (0·0%-1·8%)	174	2·3%	0·9% (0·0%-1·8%)
	**Schools reopening and second wave (Sep 20 - Dec 20)**	152	5·3%	2·5% (0·6%-4·4%)	152	5·3%	2·5% (0·6%-4·4%)
	**Post second wave (Jan 21 -Mar 21)**	195	21·5%	20·6% (13·6%-27·6%)	195	17·9%	18·1% (11·3%-24·9%)
	**Emergence of delta variant (Apr 21 - Jun 21)**	51	15·7%	17·6% (9·7%-25·5%)	51	13·7%	16·6% (8·9%-24·3%)
**15-18 years**	**Pre-pandemic (Oct 19 - Mar 20)**	28	0·0%	0·0%	28	0·0%	0·0%
	**First wave (Apr 20-May 20)**	82	6·1%	2·5% (0·5%-4·5%)	82	6·1%	2·5% (0·5%-4·5%)
	**Post first wave (Jun 20 - Aug 20)**	134	4·5%	4·6% (0·5%-8·7%)	134	5·2%	5·1% (1·0%-9·3%)
	**Schools reopening and second wave (Sep 20 - Dec 20)**	116	13·8%	23·2% (17·4%-29·0%)	117	9·4%	20·7% (15·3%-26·0%)
	**Post second wave (Jan 21 -Mar 21)**	189	20·1%	19·5% (13·8%-25·1%)	189	18·0%	17·0% (11·5%-22·4%)
	**Emergence of delta variant (Apr 21 - Jun 21)**	39	30·8%	32·7% (23·6%-41·8%)	39	28·2%	21·2% (12·1%-30·4%)

Table 8 SARS-CoV-2 seroprevalence (RocheS - Anti-SARS-CoV-2 IgG spike protein antibodies and RocheN - Anti-SARS-CoV-2 IgG nucleocapsid antibodies) by region and time period October 2019 to June 2021 in England, adjusted for age and ethnicity·

		RocheS			RocheN		
		N	Crude	Adjusted	N	Crude	Adjusted
Pre-pandemic (Oct 19 - Mar 20)	**East of England**	21	0·0%	0·0%	21	0·0%	0·0%
	**London**	10	0·0%	0·0%	10	0·0%	0·0%
	**Midlands**	0	0·0%	0·0%	0	0·0%	0·0%
	**North East & Yorkshire**	2	0·0%	0·0%	2	0·0%	0·0%
	**North West**	0	0·0%	0·0%	0	0·0%	0·0%
	**South East**	92	0·0%	0·0%	92	0·0%	0·0%
	**South West**	0	0·0%	0·0%	0	0·0%	0·0%
First wave (Apr 20 - May 20)	**East of England**	16	6·3%	3·3% (0·0%-9·3%)	16	6·3%	0·0%
	**London**	55	12·7%	10·2% (3·0%-17·3%)	55	12·7%	10·2% (3·0%-17·3%)
	**Midlands**	27	0·0%	0·0%	27	0·0%	0·0%
	**North East & Yorkshire**	64	3·1%	3·8% (0·0%-8·4%)	64	3·1%	3·8% (0·0%-8·4%)
	**North West**	0	0·0%	0·0%	0	0·0%	0·0%
	**South East**	134	2·2%	1·5% (0·0%-3·1%)	133	2·3%	1·5% (0·0%-3·1%)
	**South West**	39	0·0%	0·0%	39	0·0%	0·0%
Post first wave (Jun 20- Aug 20)	**East of England**	9	0·0%	0·0%	9	0·0%	0·0%
	**London**	14	21·4%	33·9% (18·7%-49·1%)	14	21·4%	33·9% (18·7%-49·1%)
	**Midlands**	46	2·2%	0·9% (0·0%-2·7%)	46	2·2%	0·9% (0·0%-2·7%)
	**North East & Yorkshire**	73	0·0%	0·0%	73	0·0%	0·0%
	**North West**	127	6·3%	5·8% (1·9%-9·7%)	125	6·4%	5·9% (2·0%-9·8%)
	**South East**	97	0·0%	0·0%	94	0·0%	0·0%
	**South West**	39	0·0%	0·0%	39	0·0%	0·0%
Schools reopening and second wave (Sep 20- Dec 20)	**East of England**	11	0·0%	0·0%	11	0·0%	0·0%
	**London**	12	16·7%	8·4% (2·0%-14·7%)	12	16·7%	8·4% (2·0%-14·7%)
	**Midlands**	77	6·5%	2·6% (0·1%-5·0%)	75	5·3%	5·2% (0·1%-10·3%)
	**North East & Yorkshire**	62	11·3%	11·6% (5·7%-17·4%)	61	9·8%	10·5% (5·0%-16·0%)
	**North West**	61	11·5%	15·1% (9·5%-20·7%)	61	11·5%	15·4% (9·3%-21·4%)
	**South East**	127	3·9%	3·8% (0·7%-6·9%)	122	4·1%	3·0% (0·2%-5·8%)
	**South West**	114	5·3%	5·1% (0·0%-10·2%)	114	5·3%	4·6% (0·0%-9·6%)
Post second wave (Jan 21 - Mar 21)	**East of England**	88	9·1%	13·2% (8·9%-17·5%)	76	10·5%	11·2% (7·7%-14·8%)
	**London**	115	26·1%	24·1% (15·1%-33·1%)	115	26·1%	22·8% (13·9%-31·8%)
	**Midlands**	49	14·3%	15·5% (5·4%-25·6%)	48	14·6%	15·5% (5·4%-25·6%)
	**North East & Yorkshire**	193	16·1%	14·8% (9·9%-19·7%)	187	16·6%	12·4% (7·7%-17·0%)
	**North West**	116	28·4%	26·8% (18·0%-35·7%)	115	27·8%	24·0% (15·4%-32·6%)
	**South East**	120	10·8%	11·5% (5·3%-17·8%)	109	11·0%	11·6% (5·3%-17·9%)
	**South West**	90	14·4%	18·5% (10·8%-26·2%)	89	14·6%	16·4% (9·1%-23·7%)
	**East of England**	26	7·7%	2·0% (0·0%-5·8%)	23	4·3%	2·0% (0·0%-5·8%)

Ratcliffe H, *et al. Arch Dis Child* 2023; 108:123–130. doi: 10.1136/archdischild-2022-324375

Emergence of delta variant (Apr 21 - Jun 21)	**London**	83	24·1%	25·8% (13·8%-37·8%)	83	24·1%	20·6% (9·7%-31·5%)
	**Midlands**	11	18·2%	11·3% (0·7%-21·9%)	11	18·2%	11·3% (0·7%-21·9%)
	**North East & Yorkshire**	25	24·0%	23·7% (20·3%-27·0%)	25	24·0%	7·8% (4·8%-10·9%)
	**North West**	27	40·7%	51·9% (33·4%-70·5%)	26	42·3%	51·9% (33·4%-70·5%)
	**South East**	63	11·1%	6·0% (0·1%-11·9%)	57	10·5%	5·6% (0·0%-11·5%)
	**South West**	31	16·1%	26·6% (22·0%-31·2%)	31	16·1%	28·9% (23·3%-34·6%)

Figure 2 Summary of symptoms reported by seropositive participants on RocheN Anti-SARS-CoV nucleocapsid antibodies assay by age group· Error bars show 95% confidence intervals

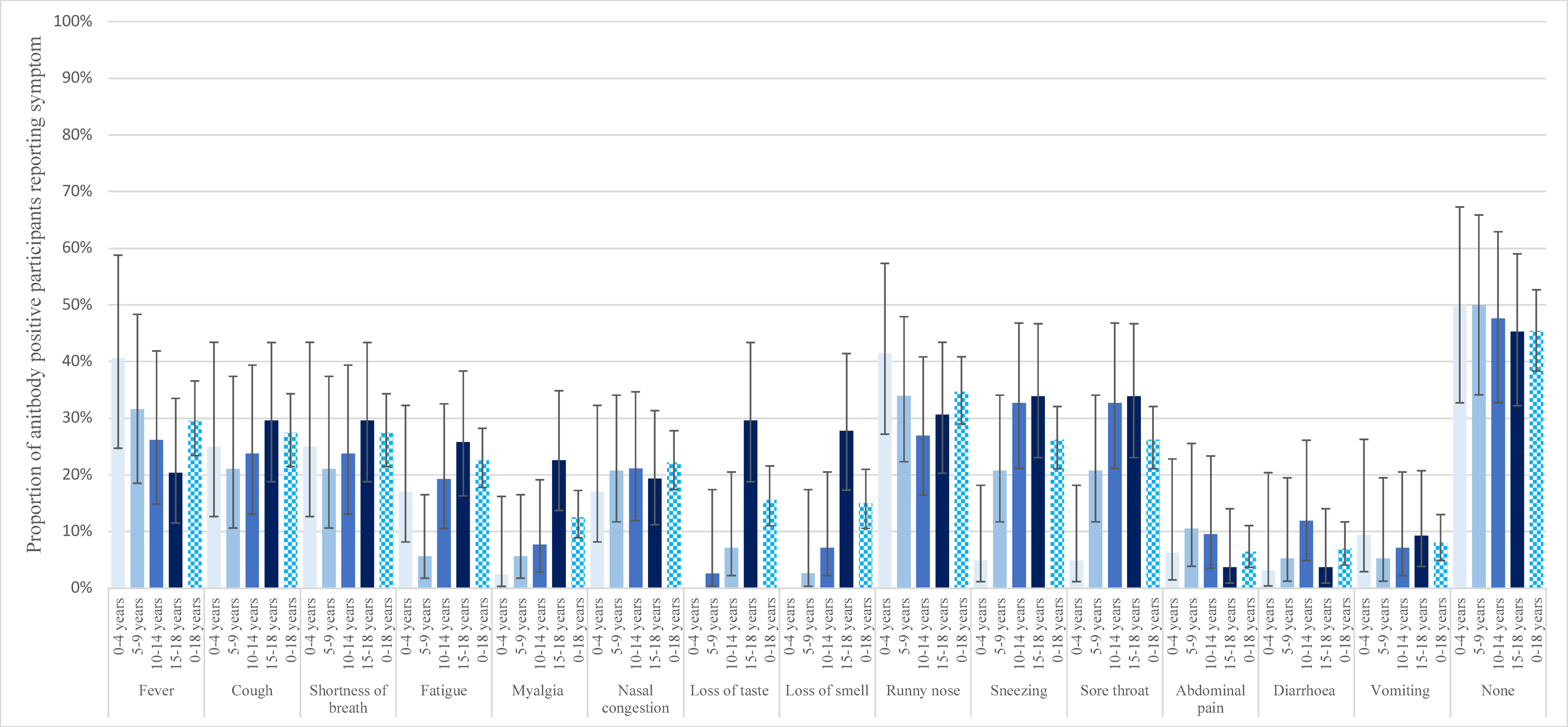

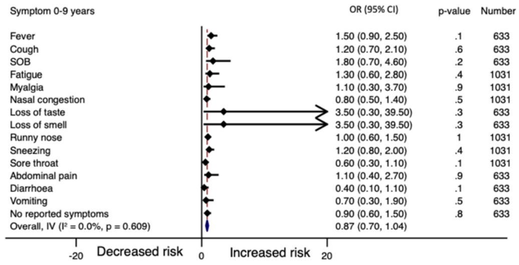

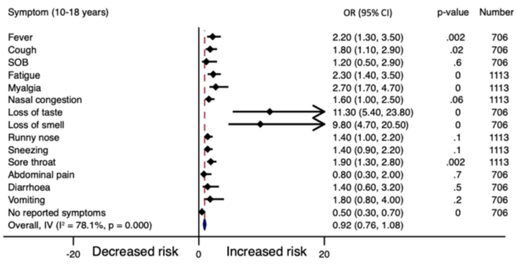

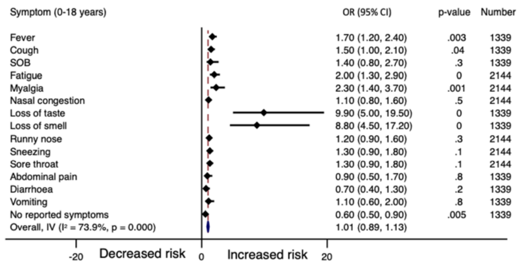

Figure 3a Univariate analysis of symptoms by age band (RocheS - Anti-SARS-CoV-2 IgG spike protein antibodies)

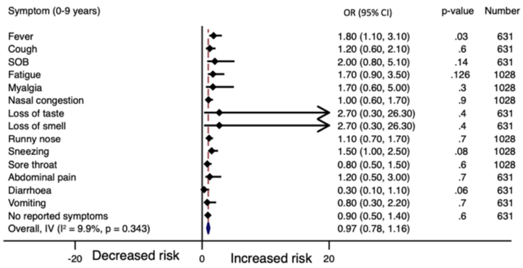

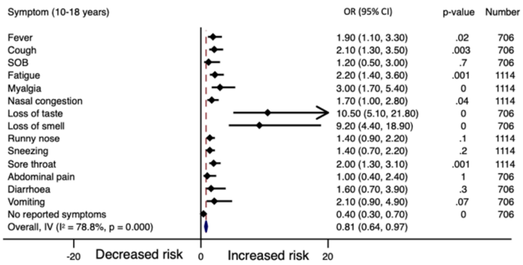

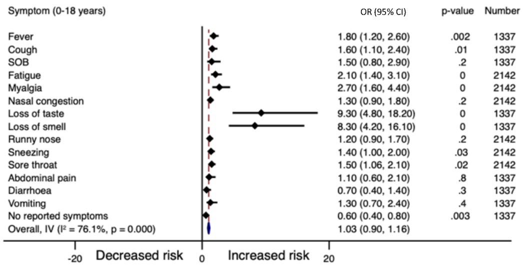

Figure 3b Univariate analysis of symptoms by age band (RocheN - Anti-SARS-CoV-2 IgG nucleocapsid antibodies)

Ratcliffe H, *et al. Arch Dis Child* 2023; 108:123–130. doi: 10.1136/archdischild-2022-324375

Table 9a Logistic regression models to determine symptoms associated with positivity the RocheS - Anti-SARS-CoV-2 IgG spike protein antibodies assay results. Symptoms with the highest p-value were sequentially excluded and model Akaike Information Criterion (AIC) values were compared until a model with the lowest AIC value had been reached. Separate models created for 0-9 years and 10-18 years.

RocheS		OR (95% CI)	p-value
**Age group: 0-9 years (N=663 observations)**			
	**Male**	1·0 (0·6-1·7)	0·89
	**Fever**	1·7 (1·0-2·9)	0·05
	**Gastrointestinal symptoms**	0·6 (0·3-1·1)	0·09
**Age group: 10-18 years (N=873 observations)**			
	**Male**	0·9 (0·6-1·3)	0·57
	**Fever**	1·6 (1·0-2·7)	0·05
	**Loss of taste and smell**	9·2 (5·1-16·3)	<0·001

OR: odds ratio

Table 9b Logistic regression models to determine symptoms associated with positivity the RocheN - Anti-SARS-CoV-2 IgG nucleocapsid antibodies assay results· Symptoms with the highest p-value were sequentially excluded and model Akaike Information Criterion (AIC) values were compared until a model with the lowest AIC value had been reached· Separate models created for 0-9 years and 10-18 years

RocheN	OR (95% CI)	p-value
**Age group: 0-9 years (N=631 observations)**		
**Male**	1·1 (0·6-1·8)	0·80
**Fever**	2·1 (1·2-3·6)	0·009
**Gastro intestinal symptoms**	0·5 (0·3-1·1)	0.09
**Age group: 10-18 years (N=873 observations)**		
**Male**	0·9 (0·6-1·4)	0·65
**Cough**	1·6 (1·0-2·6)	0·07
**Loss of taste or smell**	8·0 (4·5-14·4)	<0·001

OR: odds ratio

Ratcliffe H, *et al. Arch Dis Child* 2023; 108:123–130. doi: 10.1136/archdischild-2022-324375

Table 10 A univariate and multivariable logistic regression models to establish risk of SARS-CoV-2 seropositivity on Roche Elecsys Anti-SARS-CoV-2 serological assays for the detection of anti-SARS-CoV-2 IgG nucleocapsid antibodies (RocheN) in children aged 0-18 years·

	Univariate		Multivariable					
			IMD* deprivation quintiles		IDACI** deprivation quintiles		IMD and incl· HCW†	
**Number of participants in model**			2285		2285		1886	
	**Odds ratio (95%CI)**	**LR test (p value)**	**Odds ratio (95% CI)**	**LR test‡ (p value)**	**Odds ratio (95% CI)**	**LR test (p value)**	**Odds ratio (95% CI)**	**LR test (p value)**
**Age group**								
**0-4 years**	1·0 (0·7-1·6)	0·1	0·8 (0·5-1·3)	0·02	0·8 (0·1-1·3)	0·02	0·8 (0·5-1·3)	0·07
**5-9 years**	1·1 (0·7-1·6)		1·1 (0·7-1·7)		1·1 (0·7-1·7)		1·1 (0·7-1·6)	
**10-14 years**	1 (ref)		1 (ref)		1 (ref)		1 (ref)	
**15-18 years**	1·5 (1·1-2·2)		1·6 (1·1-2·4)		1·6 (1·1-2·4)		1·5 (1·0-2·2)	
**Sex**								
**Female**	1 (ref)		1 (ref)		1 (ref)		1 (ref)	
**Male**	0·9 (0·7-1·2)	0·7	1·0 (0·7-1·3)	0·9	1·0 (0·7-1·3)	0·9	1·0 (0·7-1·3)	0·9
**NHS region**								
**East of England**	1·1 (0·5-2·6)	<0·001	0·8 (0·3-2·0)	<0·001	0·8 (0·3-2·0)	0·001	0·9 (0·3-2·2)	0·001
**London**	5·5 (3·3-8·9)		2·7 (1·6-4·7)		2·6 (1·5-4·4)		2·7 (1·5-4·8)	
**Midlands**	1·7 (0·8-3·2)		1·3 (0·6-2·6)		1·2 (0·6-2·5)		1·2 (0·6-2·6)	
**North East and Yorkshire**	2·3 (1·3-3·8)		1·5 (0·9-2·7)		1·5 (0·8-2·6)		1·5 (0·8-2·7)	
**North West**	4·5 (2·8-7·4)		2·7 (1·5-4·9)		2·6 (1·5-4·6)		2·7 (1·4-4·9)	
**South East**	1 (ref)		1 (ref)		1 (ref)		1 (ref)	
**South West**	1·7 (0·9-3·0)		1·5 (0·8-2·7)		1·5 (0·8-2·7)		1·5 (0·8-2·7)	
**Time period**								
**Pre-pandemic (01 Oct 2019 - 31 Mar 2020)**		<0·001		<0·001		<0·001		<0·001
**First wave (01 Apr 2020 - 31 May 2020)**	0·2 (0·1-0·4)		0·2 (0·1-0·5)		0·2 (0·1-0·5)			
**Post first wave (01 Jun 2020 - 31 Aug 2020)**	0·2 (0·1-0·4)	<0·001	0·2 (0·1-0·3)		0·2 (0·1-0·3)		0·2 (0·1-0·3)	
**Schools reopening and second wave (01 Sep 2020 - 31 Dec 2020)**	0·4 (0·2-0·6)	<0·001	0·4 (0·3-0·6)		0·4 (0·2-0·6)		0·4 (0·3-0·6)	
**Post second wave (01 Jan 2021 - 31 Mar 2021)**	1 (ref)		1 (ref)		1 (ref)		1 (ref)	
**Emergence of delta variant (01 Apr 2021 - 30 Jun 2021)**	1·2 (0·8-1·7)	0·3	1·0 (0·7-1·6)		1·0 (0·7-1·6)		1·0 (0·7-1·6)	
**Ethnicity**								
**White**	1 (ref)		1 (ref)		1 (ref)		1 (ref)	
**Minority ethnic group ††**	2·5 (1·9-3·5)	<0·001	1·5 (1·0-2·1)	0·04	1·5 (1·0-2·1)	0·04	1·4 (1·0-2·1)	0·06
**IMD deprivation quintile**								

Ratcliffe H, *et al. Arch Dis Child* 2023; 108:123–130. doi: 10.1136/archdischild-2022-324375

**Most deprived 1**	2·8 (1·8-4·2)	0·002	1·5 (0·9-2·4)	0·02			1·7 (1·0-2·9)	0·009
**2**	1·4 (0·9-2·3)		0·7 (0·4-1·2)				0·7 (0·4-1·2)	
**3**	1·5 (1·0-2·3)		0·9 (0·6-1·5)				1·0 (0·6-1·7)	
**4**	1·7 (1·1-2·5)		1·4 (0·9-2·2)				1·5 (0·9-2·4)	
**Least deprived 5**	1 (ref)		1 (ref)				1 (ref)	
**IDACI deprivation quintile**								
**Most deprived 1**	2·0 (1·3-2·9)	<0·001			1·2 (0·7-1·8)	0·04		
**2**	1·0 (0·6-1·6)				0·6 (0·4-1·0)			
**3**	0·8 (0·5-1·3)				0·6 (0·4-1·0)			
**4**	1·0 (0·7-1·5)				1·0 (0·6-1·5)			
**Least deprived 5**	1 (ref)				1 (ref)			
**Urban/rural**								
**Rural**	0·2 (0·1-0·4)	<0·001	0·4 (0·2-0·8)	0·002	0·4 (0·2-0·7)	<0·001	0·5 (0·2-0·9)	0·009
**Urban**	1 (ref)		1 (ref)		1 (ref)		1 (ref)	
**HCW †**								
**No**	1 (ref)						1 (ref)	
**Yes**	1·5 (1·1-2·0)	0·003					1·7 (1·3-2·4)	<0·001

**IMD Index of Multiple Deprivation*

***IDACI Income Deprivation Affecting Children Index (IDACI) measures the proportion of all children aged 0 to 15 living in income deprived families*

†,fewer results available for this analysis due to incomplete data in HCW (healthcare worker) field (see Table 2)

††,Minority ethnic group includes all minority groups apart from white minorities·

‡Likelihood ratio for each risk factor calculated with overall p value displayed

Ratcliffe H, *et al. Arch Dis Child* 2023; 108:123–130. doi: 10.1136/archdischild-2022-324375

Appendix 1 Questionnaire used in What's the STORY? (Serum Testing Of Representative Youngsters)

Participant Number: |__| |__| |__| |__|
Most of the questions below are taken from the UK census. They are included here to check how well the participants taking part in this study match those of the general population in your area. These data will be stored securely by your site and analysed anonymously in accordance with General Data Protection Regulation (GDPR).
Today's date: _ _ / _ __ _ / _ _ _ _

Q1. What is your child's date of birth? (month and year only)
Q2. What was their birth gender? male female
Q3. Which country was your child born in?
Q4. How many years has your child lived in the UK?
Q5. Which country were you (the Parent/Guardian) born in?
Q6. How many years have you (the Parent/Guardian) lived in the UK?
Q7. In which country/countries did your child (the participant) receive their vaccinations?

UK Other
Q8. Does your child attend a childcare setting? **(This question is for under 5 year olds only)**

Yes no
If Yes which setting? Nursery □ Childminder □ Nanny □ Other □ Please specify ____________

If the answer is yes to Q8. please indicate how many hours a week?

1-4 hours	4-8 hours	8-12 hours
12-20 hours	21-30 hours	30 + hours

The following questions is for school aged children

Q9. Is your child enrolled in full time education?
Yes No
Q10. If yes to Q9, which setting are they being educated in?
Primary school
Middle school
Secondary school
College
Homeschooling
Other □ please specify
Q11. If in full time education please provide the name of the institution___________________
or N/A □ (if N/A please move to question 14)
Q12. If enrolled at an institution, is it open? Yes no (if no please move to question 14)
Q13. If your child's institution is open are they attending? Yes no N/A
Q14. Who is your child's GP? ________________________
Q15. Name and address of surgery ____________________________
Q16. Has your child had any symptoms such as those listed below since February 2020?

Fever
Dry Cough
Shortness of breath
Fatigue
Myalgia (muscle aches)
Nasal congestion
Loss of sense of taste
Loss of sense of smell
Runny nose
Sneezing

Sore throat
Abdominal pain or cramps (not including menstrual cramps)
Diarrhoea
Vomiting
Other Please specify____________
None of the above

Q17. If yes to any of the symptoms in question 16, when was the **start** of their most recent episode of symptoms? DD/MM/2020 N/A
Q18. Has your child ever been tested for COVID-19? (throat and/or nose swab specifically for COVID-19) Yes No
Q19. Has the result of your child's throat and/or nose swab for COVID-19 been positive? Yes No N/A
Q20. What was the date of the test? DD/MM/YYYY N/A
Q21. Has your child ever been tested for COVID-19 antibodies (blood test/saliva test)? Yes No
Q22. If yes to Q21, has the result of your child's antibody testing been positive?
Q23. Yes No N/A
Q24. What was the date of the antibody test? DD/MM/YYYY N/A

The following questions refer to any household contacts. A household contact is defined as a person who stays overnight in the same residence the participant.

Q25. Has anyone in your child's household(s) had a new loss of taste or smell since February 2020? Yes No
Q26. If yes to question 26, how many people in your child's household been affected? …………..
Q27. If yes to question 26 when was the ***start*** of their symptoms? DD/MM/2020
Q28. If yes to question 26, how old are they?
Q29. Has anyone in your child's household had any symptoms listed below since February 2020?

Fever
Dry cough
Shortness of breath
Muscle aches
Feeling tired
Loss of appetite
Abdominal pain or cramps (not including menstrual cramps)
Diarrhoea
Vomiting
Other Please specify_____________
None of the above

Q30. If yes to question 28, how many people in your child's household been affected? …………..
Q31. If yes to any symptoms in question 28, when was the start of their most recent episode of symptoms? DD/MM/ YYYY If yes to any symptoms in question 28, how old are they?
Q32. If yes to any symptoms in question 28 have they ever been tested for COVID-19? (throat and/or nose swab specifically for COVID-19) Yes No
Q33. If yes to any symptoms in question 28 have they ever been diagnosed with laboratory confirmed throat and/or nose swab for COVID-19? Yes No
Q34. If yes to question 33, when was their positive test? DD/MM/ YYYY Has the individual in question ever been tested for COVID-19 antibodies (blood test/saliva test)? Yes No N/A
Q35. If yes to Q35, has the result of the individual's antibody testing been positive?
Q36. Yes No N/A
Q37. What was the date of the antibody test? DD/MM/ YYYY N/A
Q38. Has anyone in the household without symptoms been tested for COVID-19? (throat and/or nose swab specifically for COVID-19) Yes No
Q39. If yes to Q38 was it positive? Yes No

Q40. What was the date of the test? DD/MM/ YYYY N/A

Q41. Has anyone in the household without symptoms been tested for COVID-19 antibodies (blood test/saliva test)? Yes No N/A

Q42. If yes to Q40 was it positive? Yes No

Q43. What was the date of the antibody test? DD/MM/ YYYY N/A

Q44. Which of the following best describes **your child? (the participant)**

White

English/Welsh/Scottish/Northern Irish/ British

Irish

Gypsy or Irish Traveller

Other White background please specify ………………

Mixed/ multiple ethnic groups

White and Black Caribbean

White and Black African

White and Asian

Any other mixed/ multiple ethnic background please specify

Asian/ Asian British

Indian

Pakistani

Bangladeshi

Chinese

Any other Asian background please specify

Black/ African/ Caribbean/Black British

African

Caribbean

Any other Black/ African/ Caribbean background please specify

Other ethnic group

Arab

Any other ethnic group please specify What is **your child's** (the participant) religion? (This question is optional)

No religion

Christian (including Church of England, Catholic, Protestant and all other Christian denominations)
Buddhist
Hindu
Jewish
Muslim
Sikh
Other please specify
Prefer not to say

Please complete questions 46 -59 for the main household in which your child lives?

Q45. How many adults (16+ years old)?

Q46. How many children (under the age of 16)?

Q47. How would you describe the main household?

A whole house or bungalow that is:
Detached
Semidetached
Terraced (including end-terrace)
A flat, maisonette or apartment that is:
In a purpose built block of flats or tenement
Part of a converted or shared house (including bedsits)
In a commercial building (for example, in an office building, hotel, or over a shop)
A mobile or temporary structure
A caravan or other mobile or temporary structure

Q48. Is this household's accommodation self-contained?

This means that all the rooms, including the kitchen, bathroom and toilet, are behind a door that only this household can use:

Yes, all the rooms are behind a door that only this household can use
No

Q49. How many rooms are available for use only by this household?

Do **NOT** count
Bathrooms
Toilets
Halls or landing
Rooms that can only be used for storage such as cupboards
Count all other rooms e.g.
Kitchens
Living rooms
Utility rooms
Bedrooms
Studies
Conservatories
If two rooms have been converted into one, count them as one room
Number of rooms ……………………………………

How many of these rooms are bedrooms?Include all rooms built or converted for use as bedrooms even if they are not currently used as bedrooms

Number of rooms………………………………………………………………..

Q50. Which of the following best describes your child's household's current accommodation?

Own you own home outright
Own your home with a mortgage
Renting from the council
Renting from a housing association
Renting from a private landlord
In shared accommodation with a housing association
Living with relatives
In housing tied to your job
Lodging within another household
Other Please specify

Q51. Your employment – Parent or Guardian to answer (please select only one as your main form of employment)

Working full time

Working part time
Unemployed
Retired
In full time or further education
Claiming Job Seekers Allowance
Incapacity Benefit
Other

Q52. Does anyone in the household work in either social care or health care?
Yes
No

Thinking about your child's immediate family (parents and siblings in the same household) please can you tell us the following?

Q53. Does your family own a car, van or truck (include company cars or vans available for private use)?
None
One
Two
Three
Four or more

Q54. Does your child have their own bedroom to themselves?
No
Yes

Q55. How many computers does your family own? (including laptops and tablets, not including games consoles and smartphones)
None
One
Two
More than two

Q56. How many bathrooms (room with a bath/shower or both) are in your home?
None
One
Two
More than two

Q57. Does your family have a dishwasher at home?
No
Yes

Q58. How many times did you travel out of the UK for a holiday/vacation last year either alone or with friends/family (in the 12 months preceding lockdown for COVID-19)?
Not at all
Once
Twice
More than Twice

Table 10 STORY research team

Name	Research Group
K. Bell	Newcastle NHS Trust
C. Kennedy	Newcastle NHS Trust
A. Bell	Newcastle NHS Trust
C. L. Coates	Newcastle NHS Trust
S. Crulley	Newcastle NHS Trust
A. Davies	Newcastle NHS Trust

Ratcliffe H, *et al. Arch Dis Child* 2023; 108:123–130. doi: 10.1136/archdischild-2022-324375

S. King	Newcastle NHS Trust
D.T.J. Metcalfe	Newcastle NHS Trust
C. Reigan	Newcastle NHS Trust
D.T.J. Fabian	Newcastle NHS Trust
R.A. Sarjeant	Newcastle NHS Trust
C. Smith	Newcastle NHS Trust
L.B. Baxter	Newcastle NHS Trust
E. Thompson	Newcastle NHS Trust
R. Wane	Bradford Children's Research Team
R. Swingler	Bradford Children's Research Team
C. Bass-Woodcock	Bradford Children's Research Team
L. Ingram	Bradford Children's Research Team
J. Pearce	St George's Vaccine Institute, St George's University Hospital NHS Trust/St George's University of London
C. Hultin	St George's Vaccine Institute, St George's University Hospital NHS Trust/St George's University of London
J.I. Pereira	St George's Vaccine Institute, St George's University Hospital NHS Trust/St George's University of London
H. Foife	St George's Vaccine Institute, St George's University Hospital NHS Trust/St George's University of London
P. Fox	St George's Vaccine Institute, St George's University Hospital NHS Trust/St George's University of London
E. Stefanova	St George's Vaccine Institute, St George's University Hospital NHS Trust/St George's University of London
F. Mabesa	St George's Vaccine Institute, St George's University Hospital NHS Trust/St George's University of London
S. Hawkins	Oxford Vaccine Group
V.H. Murphy	Oxford Vaccine Group
J. Muller	Oxford Vaccine Group
R. Cooper	Oxford Vaccine Group
R. White	Oxford Vaccine Group
S. Koleva	Oxford Vaccine Group
D.T.J. Kerr	Oxford Vaccine Group
R. Drake-Brockman	Oxford Vaccine Group
J. McEwan	Oxford Vaccine Group
H. Roberts	Oxford Vaccine Group
K. O'Brien	Oxford Vaccine Group
A. Yao	Oxford Vaccine Group
H. Trari Belhadef	Oxford Vaccine Group
H. Robinson	Oxford Vaccine Group
S. Roberts	Bristol Vaccine Centre (BVC)

E.B. Burch	Bristol Vaccine Centre (BVC)
S. Thomson-Hill	Bristol Vaccine Centre (BVC)
K. Jahans-Baynton	Bristol Vaccine Centre (BVC)
Z.B. Jordan	Bristol Vaccine Centre (BVC)
D. Ellis	Leeds Teaching Hospitals NHS Trust
R. Lidstone-Scott	Leeds Teaching Hospitals NHS Trust
E. Storr	Plymouth NHS trust
A. Carney	Plymouth NHS trust
S. Sharman	Plymouth NHS trust
N.J. Oldfield	University of Nottingham Health Service
S. Royal	University of Nottingham Health Service
S. Belton	University of Nottingham Health Service
D. Hammersley	University of Nottingham Health Service
J. Wilson	Royal Manchester Children’s Hospital
N. Philips	Royal Manchester Children’s Hospital
F. Jennings	Royal Manchester Children’s Hospital
I. Mayor	Royal Manchester Children’s Hospital
K. Wilkins	Royal Manchester Children’s Hospital
S. Williams	Royal Manchester Children’s Hospital
S. Akter	Royal Manchester Children’s Hospital
E. Ashworth	Royal Manchester Children’s Hospital
H. Dalgleish	Royal Manchester Children’s Hospital
A. Wheeler	Royal Manchester Children’s Hospital
S. Persand	Imperial College London
S.J. Burrell	Imperial College London
R. Harrison	Sheffield Children’s Hospital NHS Trust
S.J. Hill	Sheffield Children’s Hospital NHS Trust
S. Gormley	Sheffield Children’s Hospital NHS Trust
R. Owens	University Hospital Southampton NHS Foundation Trust and University of Southampton.
P.S. Munro	University Hospital Southampton NHS Foundation Trust and University of Southampton.
